# DON’T SAY GAY: LGBTQIA+ INCLUSION ON LONG-TERM CARE AND ASSISTED LIVING FACILITIES’ WEBPAGES

**DOI:** 10.1093/geroni/igad104.2848

**Published:** 2023-12-21

**Authors:** Julie Maier, Andrew Pickett

**Affiliations:** Indiana University-Bloomington, Indianapolis, Indiana, United States; Indiana University-Bloomington, Bloomington, Indiana, United States

## Abstract

Many LGBTQIA+ older adults experience a variety of health disparities resulting from a lifetime of minority stress. Due to experiences of discrimination, isolation, and stigmatization, LGBTQIA+ populations have higher rates of depressive symptomology and anxiety, as well as substance abuse when compared to heterosexual, cis-gender peers. They are also more likely to experience social isolation and have a lower average socioeconomic status. For older LGBTQIA+ adults who depend on long-term care, relative levels of facility inclusiveness have the potential to mitigate or exacerbate poor health outcomes. This study examines public signals of LGBTQIA+ inclusion by assisted living and long-term care facilities in the state of Indiana. Data was collected from a stratified-random sample of 100 long term care facilities, located in rural (n = 14), mixed (n = 24), and urban (n = 62) counties in Indiana. Each facility’s website was reviewed for the presence of LGBTQIA+ inclusion, operationalized as inclusive images, symbols, messages, anti-discrimination policies, and equal opportunity employment statements or logos. The most observed signals were statements or logos indicating non-discrimination, including fair housing policies (59%) and equal opportunity employment messages (32%). These policies were most common among facilities that were part of a larger ownership group. Non-policy LGBTQIA+ inclusion symbols were rare (1%) and included a blog post with tips on finding an LGBTQIA+ inclusive care community. Findings suggest an overall dearth of LGBTQIA+ inclusive signals among older adult care facilities in Indiana.

